# Draft Genome Assembly for the Tibetan Black Bear (*Ursus thibetanus thibetanus*)

**DOI:** 10.3389/fgene.2020.00231

**Published:** 2020-04-02

**Authors:** Chenglong Zhu, Wenjie Xu, Jianchuan Li, Chang Liu, Mingliang Hu, Yuan Yuan, Ke Yuan, Yijiuling Zhang, Xingzhi Song, Jin Han, Xinxin Cui

**Affiliations:** ^1^School of Ecology and Environment, Northwestern Polytechnical University, Xi'an, China; ^2^Department of Animal Resources, Tibet Plateau Institute of Biology, Lhasa, China

**Keywords:** Tibetan black bear, 10× genomic sequencing, genome assembly, annotation, evolution

## Introduction

Species in the bear genus are well-known for their adaptation to various environments; examples are the polar bear from the Arctic (Thiemann et al., 2008), the sun bear from rainforests (Linkie et al., 2007), and the Tibetan black bear, which inhabits the Qinghai-Tibet Plateau (QTP) (Liu, 2004). The QTP is known as the “Third Pole” since it is the largest and highest plateau in the world, with an average altitude of 4,500 m above sea level. Like the polar regions, the environment of the QTP is inimical to living organisms due to the low temperature and low level of oxygen. Large mammals, such as humans and taurine cattle, usually suffer from severe pulmonary hypertension when inhabiting high altitudes (Wu et al., 2007). Previous studies on indigenous species of the QTP have found many adaptive changes in the genes and pathways related to hypoxia responses, oxygen transport, etc. (Qiu et al., 2012). However, when bears arrived on the QTP and how they have been able to survive and thrive there remains largely unknown.

The Tibetan black bear (*Ursus thibetanus thibetanus*) is a subspecies of the Asiatic black bear (*Ursus thibetanus*) and is one of the four known bear species living on the QTP (Lan et al., 2017). Tibetan black bears are typical forest animals. They are omnivorous but feed mainly on plants. Their sense of smell and hearing are very sensitive. Most Tibetan black bears rely on hibernation to survive the cold and lack of food in winter (Liu, 2004). In recent years, due to the expansion of human activity and poaching, the Tibetan black bear has experienced a rapid population reduction. It is now classified as a vulnerable species by the International Union for Conservation of Nature (IUCN).

Here, we report a high-quality genome assembly for the Tibetan black bear based on 10 × genomic sequencing technology. This assembly and associated resources will serve as an important genetic resource for specialize, species protection, and the evolution of the bear genus.

## Data

**Figure 1:**
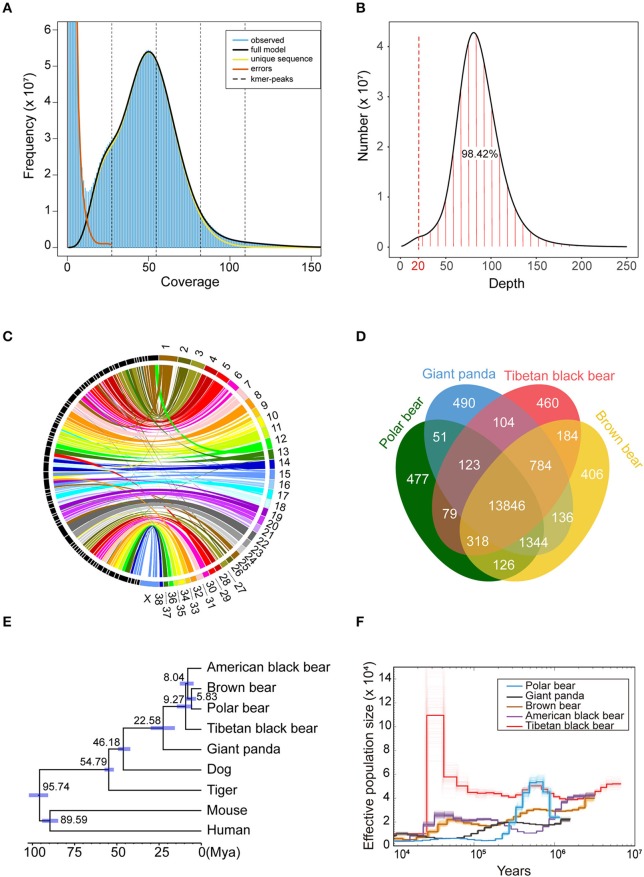
Comparative genomics of Tibetan black bear. **(A)** The input file of GenomeScope was generated by Jellyfish v2.2.6 with a k-mer size of 39. The estimated heterozygosity of the Tibetan black bear was 0.358%, and the estimated genome size was 2.36 Gb. **(B)** Distribution of sequencing depth across the whole genome assembly. A total of 98.42% of the genome sites have more than 20 coverage depths. **(C)** Conversation of syntenic relationships between the Tibetan black bear and dog chromosomes. The colored part on the right side of the picture refers to the chromosome sets of the dog genome, and the black part on the left side refers to the longest 74 scaffolds of the Tibetan black bear genome. The middle lines refer the collinearity of homology between the two species. **(D)** A Venn plot of the orthologous genes families found by OrthoFinder of four *Ursidae* species (Polar bear, Giant panda, Tibetan black bear, and Brown bear). **(E)** Phylogenetic relationships, and divergence time of nine species. The phylogenetic tree was derived from RAxML analysis, with all nodes having a bootstrap support value 100. Divergence times were estimated using MCMCtree in PAML package and are shown with 95% confidence intervals (blue bar). **(F)** Demographic history of five bear species based on PSMC software with 100 bootstraps.

The 10 × genomic sequencing technology was applied in this project. After filtering out the redundant and low-quality reads, a total of 270 Gb bases were used for the following analysis (Table 1 and Table S1). The draft genome has a size of 2.37 Gb, which is close to the estimated genome size of 2.36 Gb (Table 1, Figure 1A, Table S2). We aligned the sequenced reads with the reference genome and found that most bases (98.42%) were covered by more than 20 reads (Figure 1B). The genome assembly had the longest scaffold of 78,658,804 bp and scaffold N50 and contig N50 sizes of 26.80 Mb and 145.97 Kb, respectively (Table S2). The longest 27 and 107 scaffolds occupied 50% and 90% of all sequences, suggesting a high level of continuity.Using the mammalian gene set, BUSCO integrity assessment gave a value of 95.9% (Table S3), indicating a high level of completeness.

**Table 1:** Summary information of genome assembly and gene annotation.

**Sections**	**Results**	
**A. SEQUENCES**		
	Total bases (bp)	Total number
Raw reads	276,066,407,100	1,840,442,714
Clean reads	270,575,241,000	1,803,834,940
**B. ASSEMBLY**		
Number of scaffolds	24,973	
Total bases (bp)	2,373,600,990	
Maximum length (bp)	78,658,804	
Contig N50 (bp)	145,966	
Scaffold N50 (bp)	26,803,000	
Repeats bases (bp)	973,392,800	
**C. GENE**		
Number of genes	18,304	
Average gene length (bp)	41,420.42	
Average exon length (bp)	165.05	
Complete BUSCOs	95.40%	
Number of functional annotated genes	17,814 (97.32%)	

To further check the quality of this genome assembly, we aligned the Tibetan black bear genome with the dog (*Canis lupus familiaris*) genome (Figure 1C), which was the closest species with a chromosome-level assembly currently available. We found that some scaffolds from the Tibetan black bear genome essentially matched the dog genome at the chromosome level; for example, Scaffold00001, Scaffold00008, and Scaffold00021 corresponded to chr22, chr23, and chr31 of the dog genome assembly, respectively. We also observed evidence for several potential chromosome rearrangement events in the Tibetan black bear, including chr13 and chr28 in the dog, which appeared to lie together in the Tibetan black bear genome (Table S4).

About 41.01% of the genome assembly was estimated to be repetitive sequences, a value similar to those in the panda and dog, and LINEs occupied the largest percentage (25.02%) of repeat regions (Table S5). Using a combination of *de novo* prediction and homologous prediction, we found a total of 18,304 protein-coding genes (Table 1); of these, a total of 17,814 (97.32%) genes were found to have functional annotation in at least one of the databases mentioned above (Table S6). We compared our gene set with those from other species with respect to average gene length, average CDS length, average exon number, average exon length, and average intron length; all results were comparable to those from other species (Table S7). The BUSCO results also indicated the completeness of our gene set, with a BUSCO value of 95.4% (complete = 95.4%, single = 94.0%, duplicated = 1.4%, fragmented = 2.6%, missed = 2.0%, genes = 4,104).

We compared the gene sets of the Tibetan black bear, panda, polar bear, and brown bear and identified a total of 18,927 gene families. Within them, we found 460 species-specific genes in the Tibetan black bear, which was similar to the numbers in other bear species (Figure 1D). Using 5,229 1:1 orthologous genes between nine species, we reconstructed their phylogenetic relationships (Figure 1E). The Tibetan black bear was found to be an outgroup relative to the American black bear, the brown bear, and the polar bear, indicating a paraphyletic relationship for the black bear species, which is in concordance with a previous study (Kumar et al., 2017). The divergence time between the Tibetan black bear and the common ancestor of the polar bear, brown bear, and American black bear was estimated to take place at 9.27 million years ago (Figure 1E). Here, we found that the population history of the polar bear and American black bear was consistent with the previous research (Miller et al., 2012). Interestingly, we found that Tibetan black bear once experienced a significant population growth compared to other bear species after 100,000 years ago (Figure 1F).

## Materials and Methods

### Sample Collection, Library Construction, and Sequencing

We obtained muscle samples from a naturally dying female Tibetan black bear in the Lang county of Tibet, which has an average elevation of about 3,000 m, in 2018. The samples were thoroughly ground, and high molecular weight DNA was isolated using a RecoverEase DNA Isolation Kit (Agilent, PN 720203, San Clara, USA) with minor modifications to the manufacturer's protocol. We then followed the standard process for construction of a Chromium library. The 150 bp pair-end DNA nanospheres used for sequencing were prepared according to a previously published protocol (Drmanac et al., 2010), and the standard cyclic amplification procedure was performed on a NovaSeq 6000. The final raw sequencing file was converted to fastq format using the Supernova v2.0.0 software package (Weisenfeld et al., 2017) with default parameters for subsequent analysis (Figure S1).

### Genome Size Estimation and Genome Assembly

To predict the size of the Tibetan black bear genome, we firstly used Jellyfish v2.2.6 (Marçais and Kingsford, 2011) to count the kmer frequency with a kmer size of 39. GenomeScope web tools (parameters: Kmer length 39, Read length 150) (Vurture et al., 2017) were employed to predict the genome size, and the resulting value was around 2.36 Gb. The draft genome was assembled by the Supernova v2.0.0 software using the recommended pipeline (parameters: mkoutput -style = pseudohap - minsize = 500). Burrows-Wheeler Aligner (BWA, v0.7.15) (Li and Durbin, 2009) was used to confirm the single-base accuracy of the draft assembly with default parameters. A Benchmarking Universal Single-Copy Orthologs (BUSCO, v3.0.2) (Simao et al., 2015) analysis was applied to evaluate the completeness of the gene set in our draft genome with the library “*mammalia_odb9*”.

### Conversation of Syntenic Relationship With Dog Genome

To validate the completeness of our assembly, we compared our scaffolds to the dog chromosome set (GCF_000002285.3)^1^. Specifically, those 74 Tibetan black bear scaffolds at least 10 Mbp in length (total length ~1.92 Gb) were aligned to the dog chromosomes using LAST v942 (parameters: -E0.05) (Kielbasa et al., 2011). The best one-to-one sequence alignment for each was identified and extracted with a length of at least 10 Kb. The start and end positions of the 74 matching scaffolds that fit these criteria were then used to generate a CIRCOS plot (http://circos.ca/, accessed at Mar. 18, 2018) (Krzywinski et al., 2009), which showed regions of collinearity as well as rearrangements.

### Genome Annotation

A combined approach using *de novo* and homology-based prediction was carried out to annotate our draft genome (Wang et al., 2019). RepeatModeler v1.0.8 (http://www.repeatmasker.org/RepeatModeler, accessed at Jan. 31, 2015) was used to construct the *de novo* repeat library, and RepeatMasker v3.3.0 (Tarailo-Graovac and Chen, 2009) was employed to produce a homolog-based repeat library with default parameters. Tandem Repeats Finder v4.07 (Benson, 1999) was used to search for tandem repeat elements (parameters: 2 7 7 80 10 50 500 -d -h -ngs).

A soft-masked genome was prepared using RepeatMasker. We firstly used Augustus v3.2.1 (parameters: -species=human) (Stanke et al., 2008), GeneID v1.4.4 (parameters: -3 -P human3isoU12.param) (Alioto et al., 2018), and GlimmerHMM v3.0.3 (parameters: default) (Majoros et al., 2004) software to generate a *de novo* structure gene library. We then combined protein-coding genes from five species—human (*Homo sapiens*, GCF_000001405.38)^2^, dog (*Canis lupus familiaris*, GCF_000002285.3), giant panda (*Ailuropoda melanoleuca*, GCF_000004335.2)^3^, mouse (*Mus musculus*, GCA_000001635.8)^4^, and polar bear (*Ursus maritimus*, GCF_000687225.1)^5^—to perform homology-based gene structure prediction. To begin with, proteins from all five species were aligned with our draft genome using tBLASTN (Altschul et al., 1990) to get the rough positions of homologous genes. More accurate identification of their positions was carried out by exonerate v2.2 (parameters: -model protein2genome -percent 50 -softmasktarget T) (Slater and Birney, 2005). Finally, EVidenceModeler v1.1.1 (Haas et al., 2008) was executed to integrate homologs and *de novo* predicted genes with default parameters. Afterwards, we filtered out short low-quality genes encoding no more than 50 amino acids and genes exhibiting premature termination.

Gene function annotation was performed using InterProScan v5.30-69.0 (Jones et al., 2014) to search for domains or motifs in public databases [InterPro (Mulder and Apweiler, 2007), Gene Ontology (Ashburner et al., 2000), Pfam, SUPERFAMILY, and Panther] and KAAS web tools (Moriya et al., 2007) to search in the Kyoto Encyclopedia of Genes and Genomes database (KEGG) (Kanehisa and Goto, 2000).

### Reconstruction of Phylogenetic Relationships

The gene sets from nine species—human, mouse, dog, giant panda, polar bear, tiger (*Panthera tigris*, GCF_000464555.1)^6^, brown bear (*Ursus arctos*, GCF_003584765.1)^7^, American black bear (*Ursus americanus*, GCA_003344425.1)^8^ (Srivastava et al., 2019), and Tibetan black bear—were used for gene clustering. We identified clusters for all protein-coding genes using OrthoFinder v2.3.4 (Emms and Kelly, 2015) with default parameters. A Venn plot of four *Ursidae* species was constructed using the OrthoVenn2 webserver (Xu et al., 2019).

One-to-one orthologous gene families were detected based on the gene clusters from these nine species using the result of OrthoFinder. To reconstruct the phylogenetic relationships among these animals, multiple alignments of the genes within each single-copy gene family were performed using MUSCLE 3.8.31 (Edgar, 2004), and then the Perl script pan2nal.pl (Suyama et al., 2006) was used to convert amino acid alignment to codon alignment. Only 4-fold-degenerate sites were extracted in order to reconstruct a precise phylogenetic tree. After alignment with ClustalW2 (parameters: -CONVERT -TYPE=DNA) (McWilliam et al., 2013), the phylogenetic analysis was performed in RAXML 8.2.4 (parameters: -m GTRGAMMA -f a -x 12345 -N 100 -p 12345) (Stamatakis, 2014) for each alignment. Divergence time estimations were determined using MCMCTree (part of the PAML package) (Yang, 2007) with a correlated rate clock model. The calibration time was obtained from *www.timetree.org* (accessed at May 30, 2019), which gave human/mouse, human/dog, dog/tiger, and dog/brown bear divergence times as 85–97, 91–101, 51–56, and 42–48 million years ago (Mya), respectively. The analysis was run twice to make sure that the result was reliable.

### Population History

Pairwise sequential Markovian coalescent (PSMC) analysis (Li and Durbin, 2011), based on a hidden Markov model, was used to estimate the history of effective population sizes based on genome-wide heterozygous sequence data. The BWA-MEM algorithm (BWA v0.7.15) (Li, 2013) was used to align the clean genome sequence reads to the reference genome with default parameters. Then, by using a combination of SAMtools/BCFtools (Li et al., 2009) with PSMC recommend parameters, we obtained the consensus diploid sequences. Finally, PSMC software was run with 100 bootstraps to generate the effective population history plot. We also downloaded polar bear (SRR942298)^9^, giant panda (SRR504890)^10^, American black bear (SRR518723)^11^, and brown bear (SRR935621)^12^ resequencing data from NCBI. The same pipeline was run. The generation time and substitution rate were 11 years and 1.82e-8 per site per generation, respectively (Benazzo et al., 2017).

## Data Availability Statement

The whole genome sequencing data were submitted to the NCBI Sequence Read Archive (SRA) database with accession number SRP224444^13^ and Bioproject accession PRJNA573607. The assembled draft genome of Tibetan black bear has been deposited at GenBank with accession WEIE00000000. The annotation files of Tibetan black bear have been published ^9^ ^10^ ^11^ ^12^ ^13^ on Figshare repository with https://doi.org/10.6084/m9.figshare.11787252.v1.

## Ethics Statement

Ethical review and approval was not required for the animal study because The Tibetan black bear that died of natural causes was not applicable for animal ethic. Usage of the tissue samples in our study was approved by the government of China.

## Author Contributions

XC and JH designed and supervised the project. JL, MH, YZ, and KY prepared the samples. CZ, WX, CL, XS, and YY analyzed the data. CZ, WX, and JL wrote the manuscript with other authors' help and XC and JH revised the manuscript. All authors read and approved the final manuscript.

### Conflict of Interest

The authors declare that the research was conducted in the absence of any commercial or financial relationships that could be construed as a potential conflict of interest

## Footnotes

^1^NCBI *Assembly*, https://www.ncbi.nlm.nih.gov/assembly/GCF_000002285.3 (2011).

^2^NCBI *Assembly*, https://www.ncbi.nlm.nih.gov/assembly/GCF_000001405.38 (2017).

^3^NCBI *Assembly*, https://www.ncbi.nlm.nih.gov/assembly/GCF_000004335.2 (2009).

^4^NCBI *Assembly*, https://www.ncbi.nlm.nih.gov/assembly/GCA_000001635.8 (2017).

^5^NCBI *Assembly*, https://www.ncbi.nlm.nih.gov/assembly/GCF_000687225.1/ (2014).

^6^NCBI *Assembly*, https://www.ncbi.nlm.nih.gov/assembly/GCF_000464555.1 (2013).

^7^NCBI *Assembly*, https://www.ncbi.nlm.nih.gov/assembly/GCF_003584765.1 (2018).

^8^NCBI *Assembly*, https://www.ncbi.nlm.nih.gov/assembly/GCA_003344425.1 (2018).

^9^NCBI *Sequence Read Archive*, https://www.ncbi.nlm.nih.gov/sra/SRR942298 (2015).

^10^NCBI *Sequence Read Archive*, https://www.ncbi.nlm.nih.gov/sra/SRR504890 (2012).

^11^NCBI *Sequence Read Archive*, https://www.ncbi.nlm.nih.gov/sra/SRR518723 (2015).

^12^NCBI *Sequence Read Archive*, https://www.ncbi.nlm.nih.gov/sra/SRR935621 (2015).

^13^NCBI *Sequence Read Archive*, http://identifiers.org/ncbi/insdc.sra:SRP224444 (2019).
