# Supplementary material for: Draft Genome Assembly for the Tibetan Black Bear (Ursus thibetanus thibetanus)
Source: Front Genet. 2020 Apr 2;11:231. doi: 10.3389/fgene.2020.00231 (PMC7142260; doi:10.3389/fgene.2020.00231)
Supplement: Supplementary file 1 [file Data_Sheet_1.docx]

Supplementary Material

**Figure S1. A flow chart of sequencing, assembly, annotation, and analysis process for Tibetan black bear genome**

**Table S1. Summary information for raw sequencing reads and filtered reads**

**Table S2. Genome assembly information**

**Table S3. Comparison of the completeness of genome among five bear species in this study using mammalia_odb9 BUSCOs**

**Table S4. Conversation of syntenic relationships between dog chromosomes and Tibetan black bear scaffolds**

**Table S5. Details of repeat annotation for Tibetan black bear**

**Table S6. Summary statistics for functional annotation**

**Table S7. Summary statistics for predicted genes compared with annotation of other genomes**


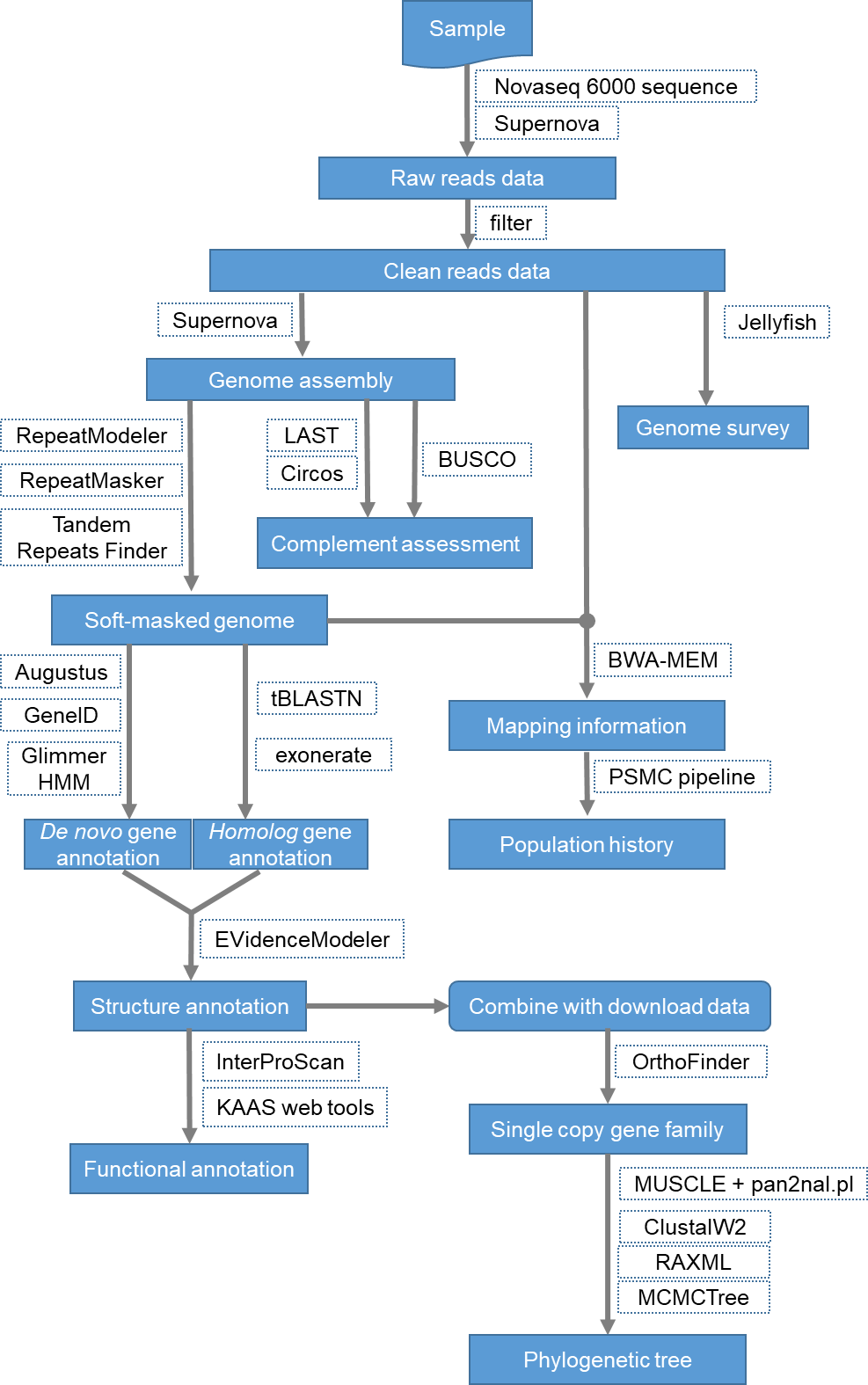


**Figure S1. A flow chart of sequencing, assembly, annotation, and analysis process for Tibetan black bear genome**

| **File** | **Raw reads** | **Raw bases (bp)** | **Clean reads** | **Clean bases (bp)** |
| --- | --- | --- | --- | --- |
| QC 0152 NDHX00177-AK831 L1 | 76,389,658 | 11,458,448,700 | 74,433,200 | 11,164,980,000 |
| QC 0152 NDHX00177-AK831 L2 | 76,094,210 | 11,414,131,500 | 74,263,848 | 11,139,577,200 |
| QC 0152 NDHX00177-AK832 L1 | 84,841,112 | 12,726,166,800 | 83,189,584 | 12,478,437,600 |
| QC 0152 NDHX00177-AK832 L2 | 84,461,972 | 12,669,295,800 | 82,936,486 | 12,440,472,900 |
| QC 0152 NDHX00177-AK833 L1 | 69,827,448 | 10,474,117,200 | 68,100,394 | 10,215,059,100 |
| QC 0152 NDHX00177-AK833 L2 | 69,541,002 | 10,431,150,300 | 67,929,434 | 10,189,415,100 |
| QC 0152 NDHX00177-AK834 L1 | 94,672,102 | 14,200,815,300 | 92,507,550 | 13,876,132,500 |
| QC 0152 NDHX00177-AK834 L2 | 94,281,492 | 14,142,223,800 | 92,274,684 | 13,841,202,600 |
| QC 0162 NDHX00177-AK831 L1 | 71,686,522 | 10,752,978,300 | 70,382,322 | 10,557,348,300 |
| QC 0162 NDHX00177-AK831 L2 | 67,959,308 | 10,193,896,200 | 66,681,870 | 10,002,280,500 |
| QC 0162 NDHX00177-AK832 L1 | 80,039,040 | 12,005,856,000 | 78,991,054 | 11,848,658,100 |
| QC 0162 NDHX00177-AK832 L2 | 75,798,244 | 11,369,736,600 | 74,779,722 | 11,216,958,300 |
| QC 0162 NDHX00177-AK833 L1 | 66,402,858 | 9,960,428,700 | 65,245,018 | 9,786,752,700 |
| QC 0162 NDHX00177-AK833 L2 | 62,781,654 | 9,417,248,100 | 61,647,500 | 9,247,125,000 |
| QC 0162 NDHX00177-AK834 L1 | 89,019,200 | 13,352,880,000 | 87,616,588 | 13,142,488,200 |
| QC 0162 NDHX00177-AK834 L2 | 84,309,840 | 12,646,476,000 | 82,938,160 | 12,440,724,000 |
| QC 0163 NDHX00177-AK831 L1 | 68,563,612 | 10,284,541,800 | 66,878,046 | 10,031,706,900 |
| QC 0163 NDHX00177-AK831 L2 | 70,553,216 | 10,582,982,400 | 68,915,118 | 10,337,267,700 |
| QC 0163 NDHX00177-AK832 L1 | 76,041,628 | 11,406,244,200 | 74,710,842 | 11,206,626,300 |
| QC 0163 NDHX00177-AK832 L2 | 78,226,438 | 11,733,965,700 | 76,947,428 | 11,542,114,200 |
| QC 0163 NDHX00177-AK833 L1 | 62,974,562 | 9,446,184,300 | 61,491,126 | 9,223,668,900 |
| QC 0163 NDHX00177-AK833 L2 | 64,958,776 | 9,743,816,400 | 63,515,136 | 9,527,270,400 |
| QC 0163 NDHX00177-AK834 L1 | 84,305,028 | 12,645,754,200 | 82,495,704 | 12,374,355,600 |
| QC 0163 NDHX00177-AK834 L2 | 86,713,792 | 13,007,068,800 | 84,964,126 | 12,744,618,900 |
| Total | 1,840,442,714 | 276,066,407,100 | 1,803,834,940 | 270,575,241,000 |

## Table S1. Summary information for raw sequencing reads and filtered reads

## Table S2. Genome assembly information

|  | **Species** | **N50** | **N60** | **N70** | **N80** | **N90** | **Longest** | **Shortest** | **Total** |
| --- | --- | --- | --- | --- | --- | --- | --- | --- | --- |
| Scaffold(bp) | Tibetan black bear | 26,803,000 | 18,859,362 | 13,463,345 | 10,191,868 | 3,842,341 | 78,658,804 | 500 | 2,373,600,990 |
|  | Panda | 1,281,781 | 1,021,505 | 777,973 | 559,162 | 312,670 | 6,047,896 | 100 | 2,299,509,015 |
|  | Polar bear | 15,940,661 | 13,021,217 | 8,656,630 | 5,608,078 | 3,219,439 | 67,462,175 | 200 | 2,301,379,344 |
|  | Brown bear | 36,708,181 | 26,537,321 | 19,211,889 | 13,463,457 | 7,357,467 | 92,727,749 | 234 | 2,328,659,882 |
|  | American black bear | 189,900 | 144,626 | 102,501 | 61,920 | 18,831 | 2,352,914 | 201 | 2,588,393,918 |
| Contig(bp) | Tibetan black bear | 145,966 | 116,988 | 89,220 | 63,158 | 34,925 | 972,066 | 48 | 2,357,716,920 |
|  | Panda | 39,886 | 31,466 | 23,965 | 17,020 | 9,848 | 434,635 | 10 | 2,245,312,831 |
|  | Polar bear | 46,506 | 36,246 | 27,260 | 18,730 | 10,413 | 477,788 | 200 | 2,263,021,934 |
|  | Brown bear | 314,844 | 248,301 | 193,011 | 137,242 | 77,985 | 2,374,379 | 50 | 2,312,446,669 |
|  | American black bear | 11,705 | 9,358 | 7,240 | 5,097 | 2,772 | 141,485 | 0 | 2,451,851,856 |

## Table S3. Comparison of the completeness of genome among five bear species in this study using mammalia_odb9 BUSCOs

| **Species** | **Tibetan black bear** | **American black bear** | **Polar bear** | **Brown bear** | **Giant panda** |
| --- | --- | --- | --- | --- | --- |
| Total number of matches to ortholog | 95.90% | 86.30% | 94.70% | 96.10% | 95.20% |
| Complete one-to-one match to ortholog | 94.40% | 85.70% | 94.30% | 95.20% | 94.90% |
| Complete match of multi-gene copies to ortholog | 1.50% | 0.60% | 0.40% | 0.90% | 0.30% |
| Fragmented match to ortholog | 1.80% | 9.10% | 3.00% | 1.90% | 2.60% |
| No match to ortholog | 2.30% | 4.60% | 2.30% | 2.00% | 2.20% |

## Table S4. Conversation of syntenic relationships between dog chromosomes and Tibetan black bear scaffolds

| **Dog chromosome** | **Number** | **Tibetan black bear Scaffolds** | | | | | | | |
| --- | --- | --- | --- | --- | --- | --- | --- | --- | --- |
| chr22 | 1 | Scaffold00001 |  |  |  |  |  |  |  |
| chr23 | 1 | Scaffold00008 |  |  |  |  |  |  |  |
| chr31 | 1 | Scaffold00021 |  |  |  |  |  |  |  |
| chr26 | 2 | Scaffold00047 | Scaffold00038 |  |  |  |  |  |  |
| chr14 | 2 | Scaffold00018 | Scaffold00005 |  |  |  |  |  |  |
| chr8 | 2 | Scaffold00011 | Scaffold00034 |  |  |  |  |  |  |
| chr17 | 2 | Scaffold00022 | Scaffold00016 |  |  |  |  |  |  |
| chr36 | 2 | Scaffold00004 | Scaffold00017 |  |  |  |  |  |  |
| chr28 | 2 | Scaffold00023 | Scaffold00027 |  |  |  |  |  |  |
| chr10 | 2 | Scaffold00022 | Scaffold00012 |  |  |  |  |  |  |
| chr32 | 2 | Scaffold00025 | Scaffold00003 |  |  |  |  |  |  |
| chr37 | 2 | Scaffold00004 | Scaffold00032 |  |  |  |  |  |  |
| chr29 | 2 | Scaffold00006 | Scaffold00040 |  |  |  |  |  |  |
| chr35 | 2 | Scaffold00030 | Scaffold00040 |  |  |  |  |  |  |
| chr38 | 2 | Scaffold00067 | Scaffold00042 |  |  |  |  |  |  |
| chr30 | 3 | Scaffold00045 | Scaffold00056 | Scaffold00069 |  |  |  |  |  |
| chr18 | 3 | Scaffold00026 | Scaffold00068 | Scaffold00005 |  |  |  |  |  |
| chr3 | 3 | Scaffold00009 | Scaffold00029 | Scaffold00010 |  |  |  |  |  |
| chr12 | 3 | Scaffold00020 | Scaffold00030 | Scaffold00002 |  |  |  |  |  |
| chr33 | 3 | Scaffold00021 | Scaffold00015 | Scaffold00062 |  |  |  |  |  |
| chr27 | 3 | Scaffold00070 | Scaffold00059 | Scaffold00061 |  |  |  |  |  |
| chr13 | 3 | Scaffold00006 | Scaffold00025 | Scaffold00010 |  |  |  |  |  |
| chr20 | 3 | Scaffold00072 | Scaffold00054 | Scaffold00024 |  |  |  |  |  |
| chr19 | 3 | Scaffold00001 | Scaffold00003 | Scaffold00017 |  |  |  |  |  |
| chr6 | 3 | Scaffold00013 | Scaffold00053 | Scaffold00039 |  |  |  |  |  |
| chr34 | 3 | Scaffold00071 | Scaffold00015 | Scaffold00008 |  |  |  |  |  |
| chr24 | 3 | Scaffold00049 | Scaffold00057 | Scaffold00048 |  |  |  |  |  |
| chrX | 3 | Scaffold00035 | Scaffold00074 | Scaffold00043 |  |  |  |  |  |
| chr21 | 4 | Scaffold00026 | Scaffold00036 | Scaffold00044 | Scaffold00068 |  |  |  |  |
| chr16 | 4 | Scaffold00018 | Scaffold00032 | Scaffold00065 | Scaffold00005 |  |  |  |  |
| chr25 | 4 | Scaffold00004 | Scaffold00032 | Scaffold00001 | Scaffold00003 |  |  |  |  |
| chr7 | 4 | Scaffold00033 | Scaffold00067 | Scaffold00039 | Scaffold00007 |  |  |  |  |
| chr11 | 4 | Scaffold00028 | Scaffold00031 | Scaffold00066 | Scaffold00051 |  |  |  |  |
| chr9 | 4 | Scaffold00031 | Scaffold00055 | Scaffold00060 | Scaffold00063 |  |  |  |  |
| chr1 | 5 | Scaffold00019 | Scaffold00046 | Scaffold00050 | Scaffold00002 | Scaffold00007 |  |  |  |
| chr15 | 5 | Scaffold00012 | Scaffold00013 | Scaffold00025 | Scaffold00041 | Scaffold00003 |  |  |  |
| chr4 | 5 | Scaffold00071 | Scaffold00014 | Scaffold00009 | Scaffold00027 | Scaffold00038 |  |  |  |
| chr5 | 6 | Scaffold00013 | Scaffold00019 | Scaffold00052 | Scaffold00036 | Scaffold00037 | Scaffold00058 |  |  |
| chr2 | 8 | Scaffold00019 | Scaffold00009 | Scaffold00028 | Scaffold00052 | Scaffold00041 | Scaffold00073 | Scaffold00064 | Scaffold00008 |

## Table S5. Details of repeat annotation for Tibetan black bear

| **Type** | | **Number** | **Length (bp)** | **Percent (%)** |
| --- | --- | --- | --- | --- |
| Class I | LTR | 389,058 | 126,101,966 | 5.31 |
|  | LINE | 1,550,309 | 593,952,434 | 25.02 |
|  | SINE | 939,648 | 148,636,166 | 6.26 |
| Class II | DNA Transposon | 386,805 | 74,996,585 | 3.16 |
|  | RC/Helitron | 1,736 | 370,463 | 0.02 |
| Satellite | | 1,227 | 446,524 | 0.02 |
| Low complexity | | 100,077 | 5,324,762 | 0.22 |
| Simple repeat | | 641,139 | 28,408,018 | 1.20 |
| TRF | | 437,332 | 30,709,449 | 1.29 |
| Others | | 2,062 | 243,673 | 0.01 |
| Unknown | | 16,059 | 7,981,670 | 0.34 |
| Total | | 4,465,452 | 973,392,800 | 41.01 |

## Table S6. Summary statistics for functional annotation

| **Database** | **Number** | **Percentage** |
| --- | --- | --- |
| GO | 13,049 | 71.29% |
| InterPro | 16,995 | 92.85% |
| PANTHER | 17,752 | 96.98% |
| Pfam | 16,537 | 90.35% |
| SUPERFAMILY | 13,491 | 73.71% |
| KEGG | 12,248 | 66.91% |
| Total | 17,814 | 97.32% |

## Table S7. Summary statistics for predicted genes compared with annotation of other genomes

| **Gene set** | **Numbers** | **Average Gene Length (bp)** | **Average CDS Length (bp)** | **Average Exon number** | **Average Exon Length (bp)** | **Average Intron Length (bp)** |
| --- | --- | --- | --- | --- | --- | --- |
| Brown bear | 19,560 | 40,925.70 | 1,709.85 | 9.72 | 175.88 | 4,496.39 |
| Dog | 19,414 | 43,101.15 | 1,702.57 | 9.81 | 173.50 | 4,697.46 |
| Human | 22,767 | 46,447.03 | 1,711.50 | 9.65 | 177.35 | 5,171.62 |
| Mouse | 23,440 | 37,134.84 | 1,642.86 | 9.06 | 181.32 | 4,403.18 |
| Giant panda | 18,886 | 39,248.75 | 1,664.86 | 9.82 | 169.50 | 4,260.07 |
| Polar bear | 18,571 | 39,325.01 | 1,618.41 | 9.71 | 166.60 | 4,326.83 |
| Tibetan black bear | 18,304 | 40,203.80 | 1,700.48 | 9.61 | 176.89 | 4,470.18 |
| Tiger | 17,908 | 41,420.42 | 1,647.96 | 9.98 | 165.05 | 4,426.80 |
